# Optimizing Drug Therapy in ECMO-Supported Critically Ill Adults: A Narrative Review and Clinical Guide

**DOI:** 10.3390/pharmacy13060151

**Published:** 2025-10-23

**Authors:** Abraham Rocha-Romero, Jose Miguel Chaverri-Fernandez, Fianesy Chaves-Fernández, Esteban Zavaleta-Monestel

**Affiliations:** 1 Pharmacy Department, Clínica Bíblica, San José 1307-1000, Costa Rica; arocha@clinicabiblica.com; 2 Faculty of Pharmacy, University of Costa Rica, San José 11501-2060, Costa Rica; jose.chaverri@ucr.ac.cr; 3 Pharmacy Department, Calderón Guardia Hospital, San José 494-1000, Costa Rica; fchavesf@ccss.sa.cr; 4 Health Research Department, Clínica Bíblica, San José 1307-1000, Costa Rica

**Keywords:** extracorporeal membrane oxygenation, pharmacokinetics, pharmacodynamics, critical illness, intensive care, drug dosing, therapeutic drug monitoring

## Abstract

Extracorporeal membrane oxygenation (ECMO) is increasingly used to support critically ill adults with severe cardiac or respiratory failure, but ECMO circuits and the physiological disturbances of critical illness significantly alter drug pharmacokinetics (PK) and pharmacodynamics (PD), complicating dosing and monitoring. This narrative review synthesizes current clinical evidence on ECMO-related PK/PD alterations and provides practical guidance for optimizing pharmacotherapy in adult intensive care. A structured literature search (January–May 2025) was conducted across PubMed/MEDLINE, EMBASE, Scopus, Cochrane Library, Sage Journals, ScienceDirect, Taylor & Francis Online, SpringerLink, and specialized databases, focusing on seven therapeutic classes commonly used in ECMO patients. Eligible studies included clinical trials, observational studies, systematic reviews, and practice guidelines in adults, while pediatric and preclinical data were excluded. Evidence quality varied substantially across drug classes. Hydrophilic, low-protein-bound agents such as β-lactams, aminoglycosides, fluconazole, and caspofungin generally showed minimal ECMO-specific PK alterations, with dose adjustment mainly driven by renal function. Conversely, lipophilic and highly protein-bound drugs including fentanyl, midazolam, propofol, voriconazole, and liposomal amphotericin B exhibited substantial circuit adsorption and variability, often requiring higher loading doses, prolonged infusions, and rigorous therapeutic drug monitoring. No ECMO-specific data were identified for certain neuromuscular blockers, antivirals, and electrolytes. Overall, individualized dosing guided by therapeutic drug monitoring (TDM), organ function, and validated PK principles remains essential to optimize therapy in this complex population.

## 1. Introduction

Extracorporeal membrane oxygenation (ECMO) is an advanced life support technique increasingly utilized to support patients with severe cardiac and respiratory failure. ECMO temporarily substitutes or supplements cardiopulmonary function by facilitating gas exchange, removing carbon dioxide, and enriching blood when conventional oxygen therapies, such as mechanical ventilation and pharmacological support, are insufficient [1]. Clinically, ECMO involves a closed extracorporeal circuit where blood is withdrawn via a cannula, pumped through an artificial membrane oxygenator, and subsequently returned to systemic circulation [2]. Common indications include severe acute respiratory distress syndrome (ARDS), refractory cardiogenic shock, acute heart failure, and other conditions characterized by profound respiratory or circulatory dysfunction [3,4]. Its use has significantly expanded in recent years, driven by advances in technology, refined protocols, and supportive clinical evidence.

Despite these benefits, ECMO poses significant pharmacokinetic challenges. The extracorporeal circuit comprising tubing, pumps, and membrane oxygenators can sequester medications such as antibiotics, antifungals, sedatives, analgesics, and other agents with high lipophilicity or extensive protein binding, leading to reduced plasma concentrations and potential therapeutic failure [5]. This phenomenon is primarily driven by physicochemical drug properties, particularly protein binding greater than 70% and log *p*-values above 2, which complicate dosing strategies [5]. In addition, ECMO patients frequently experience profound physiological alterations associated with critical illness, including expanded volume of distribution, hypoalbuminemia, impaired hepatic or renal clearance, and substantial fluid shifts, all of which further impair pharmacokinetic predictability [6,7]. As a result, conventional standardized dosing regimens may fail to achieve therapeutic targets, highlighting the need for individualized approaches, TDM and coordinated interdisciplinary management [5].

Pharmacokinetic alterations associated with ECMO are not uniform across age groups. Neonatal and infant studies have demonstrated distinct patterns, including maturational clearance, altered volume of distribution, and circuit-specific effects that differ substantially from older patients [8]. These age-dependent differences caution strongly against extrapolating pediatric or neonatal ECMO pharmacokinetics to adult populations. Accordingly, the present review focuses exclusively on adults, aiming to synthesize drug-specific pharmacokinetic and pharmacodynamic data in critically ill adults on ECMO, where dedicated evidence remains comparatively scarce but is essential for optimizing therapy.

Several reviews have previously addressed pharmacokinetic alterations in ECMO. Some authors provided one of the earliest structured summaries, outlining circuit sequestration, increased volume of distribution, and altered clearance as potential mechanisms [9]. However, most of the data were derived from neonatal and pediatric ECMO, with very limited adult pharmacokinetic evidence, and practical guidance was minimal. More recently, the focus antimicrobials in adult ECMO, summarizing current dosing strategies but restricting scope to anti-infectives and providing largely narrative recommendations without critical bias appraisal or standardized evidence grading [10]. Some narrative reviews expanded the scope to include sedatives, analgesics, antimicrobials, and anticoagulants, offering broad coverage but still as a narrative synthesis, without systematic quality assessment or harmonization of infusion strategies and units [5].

In contrast, the present review provides a dedicated adult-only perspective, applies the Joanna Briggs Institute (JBI) critical appraisal tools across all included studies, and introduces a structured evidence grading framework (A–D). We also try to distinguish ECMO-specific circuit effects from critical-illness or renal/Continuous Renal Replacement Therapy (CRRT)-driven variability, which is rarely made explicit in prior reviews. Furthermore, dosing recommendations are standardized with consistent units and infusion terminology (extended vs. continuous) and integrated with up-to-date data from recent Population Pharmacokinetics (PopPK) studies, therapeutic drug monitoring cohorts, and even randomized clinical trials on model-informed precision dosing. This approach allows the reader to weigh drug-specific recommendations across classes with greater transparency and clinical applicability than earlier reviews.

This review synthesizes the available evidence, summarizes dosing recommendations, and highlights existing gaps to improve pharmacotherapy in adult ECMO-supported patients.

## 2. Materials and Methods

### 2.1. Search Strategy

A comprehensive literature search was performed between January and May 2025 in PubMed/MEDLINE, EMBASE, Scopus, Cochrane Library, Sage Journals, ScienceDirect, Taylor & Francis Online, and SpringerLink. Additionally, specialized clinical databases (Micromedex, DynaMed, UpToDate) were queried to capture non-indexed clinical recommendations. The strategy combined controlled vocabulary (MeSH and Boolean operators) with free-text keywords related to “extracorporeal membrane oxygenation,” “critical illness,” and International Nonproprietary Names (INN) of commonly used intensive care medications. Database specific filters were applied. The database strategy search and filters are in Appendix A.

### 2.2. Eligibility Criteria

Inclusion criteria were as follows:

Adult patients (≥18 years) receiving ECMO support in intensive care units. Study designs: prospective or retrospective observational studies, interventional trials, systematic or narrative reviews, meta-analyses, and clinical practice guidelines. Focus on pharmacokinetics (PK), pharmacodynamics (PD), dosing strategies, or therapeutic drug monitoring (TDM). Publications in English or Spanish within the last 10 years.

Exclusion criteria were as follows:

Pediatric studies. Case reports or case series with <5 patients. Preclinical, in vitro, or ex vivo studies without direct clinical correlation. Editorials, expert opinions, or letters without original data.

### 2.3. Medication Selection

A multidisciplinary panel of intensivists and clinical pharmacists identified seven therapeutic classes frequently used in ECMO: (1) analgesics; (2) anesthetics and sedatives; (3) antimicrobials; (4) adrenergic agents; (5) anticoagulants; (6) neuromuscular blockers; and (7) electrolytes. Representative drugs within each class were chosen based on frequency of Intensive Care Unit (ICU) use, likelihood of ECMO-related PK/PD alterations, and preliminary evidence from scoping searches.

### 2.4. Data Extraction and Synthesis

Two reviewers independently screened titles and abstracts, followed by full-text review. Discrepancies were resolved by consensus. For each included drug, extracted data included study design, population, ECMO modality (veno-venous or veno-arterial), reported PK/PD alterations, dosing regimens, TDM recommendations, and clinical precautions. Findings were synthesized narratively into drug-specific summaries and compiled into a structured pharmacotherapy guide.

### 2.5. Use of Generative Artificial Intelligence

Generative artificial intelligence (ChatGPT, GPT-5, OpenAI, San Francisco, CA, USA) was used to assist in text editing, rephrasing for clarity, and formatting. In addition, ChatGPT was employed to generate preliminary schematic elements and conceptual diagrams. These outputs were subsequently modified, refined, and integrated by the authors into the final figures included in this manuscript. It was not used for data extraction, data analysis, or interpretation of study results. The authors have carefully reviewed and edited all outputs and take full responsibility for the content of this publication.

### 2.6. Risk of Bias

The methodological quality and risk of bias of all included studies were critically appraised using the Joanna Briggs Institute (JBI) Critical Appraisal Tools, applying the checklist appropriate to each study design (systematic reviews, cohort studies, case–control studies, analytical cross-sectional studies, and case series). Each tool evaluates domains related to study selection, validity of exposure and outcome measurements, identification and management of confounders, completeness of follow-up, and appropriateness of statistical analyses. Two independent reviewers independently performed the critical appraisal, assigning a judgment of Yes, No, Unclear, or Not applicable for each item. Discrepancies were resolved by consensus.

### 2.7. Evidence Grading and Target Harmonization

We assigned an A–D evidence grade to each drug based on study design and consistency (A: multicenter prospective/systematic review with consistent findings; B: prospective multicenter studies that analyses population pharmacokinetics or robust comparative cohorts; C: small single-center observational data; D: case series/reports or extrapolation). PK/PD denominators were harmonized across classes: β-lactams as %fT > MIC (with %fT > 4 × MIC for severe infections), aminoglycosides as Cmax/MIC, glycopeptides as AUC24/MIC, linezolid/azoles as trough (Cmin), and echinocandins as AUC/MIC.

## 3. Results

Our structured literature search identified a heterogeneous body of evidence spanning observational studies, systematic reviews, meta-analyses, and limited interventional investigations in adult ECMO populations. The impact of extracorporeal membrane oxygenation (ECMO) on drug pharmacokinetics (PK) and pharmacodynamics (PD) was consistently shown to depend on both circuit-related factors (e.g., oxygenator type, tubing material, and duration of circuit use) and drug-specific physicochemical properties such as protein binding, lipophilicity, molecular weight, and volume of distribution. In addition, the profound physiological disturbances associated with critical illness, including altered clearance, fluid shifts, and changes in protein concentrations were found to contribute substantially to interpatient variability. Robust, multicenter pharmacokinetic studies remain scarce, and the quality of available data varied widely across therapeutic classes, ranging from well-characterized antimicrobials to drugs with little or no ECMO-specific evidence. The following sections summarize key findings by drug class, outlining patterns of PK/PD alterations and highlighting areas where individualized dosing, therapeutic drug monitoring (TDM), and clinical judgment are essential. A detailed synthesis of dosage recommendations, PK/PD considerations, monitoring strategies, alternatives, and clinical precautions is presented in a proposed guideline with Suggest Recommendations.

### 3.1. Analgesics

Among opioids, morphine exhibited minimal ECMO-related sequestration due to its hydrophilicity, although active metabolite accumulation remains a concern in renal dysfunction [11,12,13,14]. In contrast, fentanyl demonstrated significant circuit adsorption, requiring higher doses and individualized titration [15,16,17,18]. These findings highlight the need for close monitoring of efficacy and safety, with multimodal analgesia strategies often recommended.

### 3.2. Anesthetics and Sedatives

Ketamine showed limited sequestration, with variability mostly explained by critical illness rather than ECMO [19,20,21]. Propofol and midazolam, both highly lipophilic and protein-bound, were markedly affected by circuit adsorption, frequently necessitating higher doses and raising concerns about drug accumulation and adverse effects [15,22,23,24]. Diazepam is generally discouraged due to prolonged half-life and high adsorption risk [25,26,27]. Overall, sedative management requires careful titration and, when available, TDM [28].

### 3.3. Antimicrobials

Hydrophilic agents such as β-lactams (e.g., ceftazidime, meropenem) and aminoglycosides (e.g., amikacin) displayed minimal direct ECMO effects, with dose variability mainly driven by renal function and fluid balance [29,30,31,32,33,34,35,36]. Given the high PK variability observed in ECMO patients, embedding model-informed precision dosing (MIPD) with therapeutic drug monitoring (TDM) for time-dependent antibiotics is particularly relevant. In critically ill adults, time-dependent antibiotics like β-lactams and ciprofloxacin the feasibility of Bayesian PK modeling with bedside TDM, although without improvement in ICU length of stay [37,38]. These results underscore that target attainment is challenging even in non-ECMO ICU cohorts, justifying the adoption of proactive MIPD/TDM workflows (extended or continuous infusions, systematic auditing of PK/PD targets) in ECMO, where variability is at least as great [37]. In contrast, lipophilic antifungals like voriconazole and liposomal amphotericin B showed pronounced sequestration and interpatient variability, often requiring dose escalation and rigorous TDM [39,40,41,42,43,44,45,46,47]. Agents such as linezolid and vancomycin exhibited mixed results, with individualized dosing and monitoring strongly advised [48,49,50,51,52,53,54,55,56,57,58,59,60]. For several drugs, including ganciclovir, acyclovir, and certain antivirals, no ECMO-specific clinical data were available.

### 3.4. Adrenergic Agents

Norepinephrine pharmacokinetics appeared largely unaffected by ECMO, with dose requirements reflecting illness severity rather than circuit effects [61,62,63]. Conversely, epinephrine use in VA-ECMO was associated with increased mortality and adverse outcomes, warranting restriction to refractory scenarios [64,65]. Dobutamine showed limited data, with recommendations favoring cautious use at low doses or alternatives such as milrinone or levosimendan [66].

### 3.5. Anticoagulants

Unfractionated heparin (UFH) remains the standard anticoagulant, with minimal circuit sequestration but high interpatient variability, necessitating multimodal monitoring of Anti-factor Xa (FXa), Activated Partial Thromboplastin Time (aPTT), and Activated Clotting Time (ACT) (anti-FXa, aPTT, ACT) [67,68,69,70,71,72]. Enoxaparin, used either subcutaneously or intravenously, emerged as a feasible alternative with stable PK profiles and comparable safety, although evidence remains limited [73,74]. Alternatives such as bivalirudin and argatroban may be considered in cases of heparin-induced thrombocytopenia or organ dysfunction [75,76].

### 3.6. Neuromuscular Blockers

Evidence was scarce for this class. Rocuronium was noted for potential accumulation due to its lipophilicity and hepatic metabolism, but clinical data were insufficient to define ECMO-specific dosing adjustments. Cisatracurium is generally preferred because of its organ-independent metabolism [22,77,78].

### 3.7. Electrolytes

For agents such as potassium chloride, magnesium sulfate, calcium gluconate, and potassium phosphate, no ECMO-specific pharmacokinetic data were identified. Current practice relies on standard ICU dosing with careful titration according to serum levels and organ function. These represent critical evidence gaps that warrant further research.

### 3.8. Study Selection

**Figure 1:**
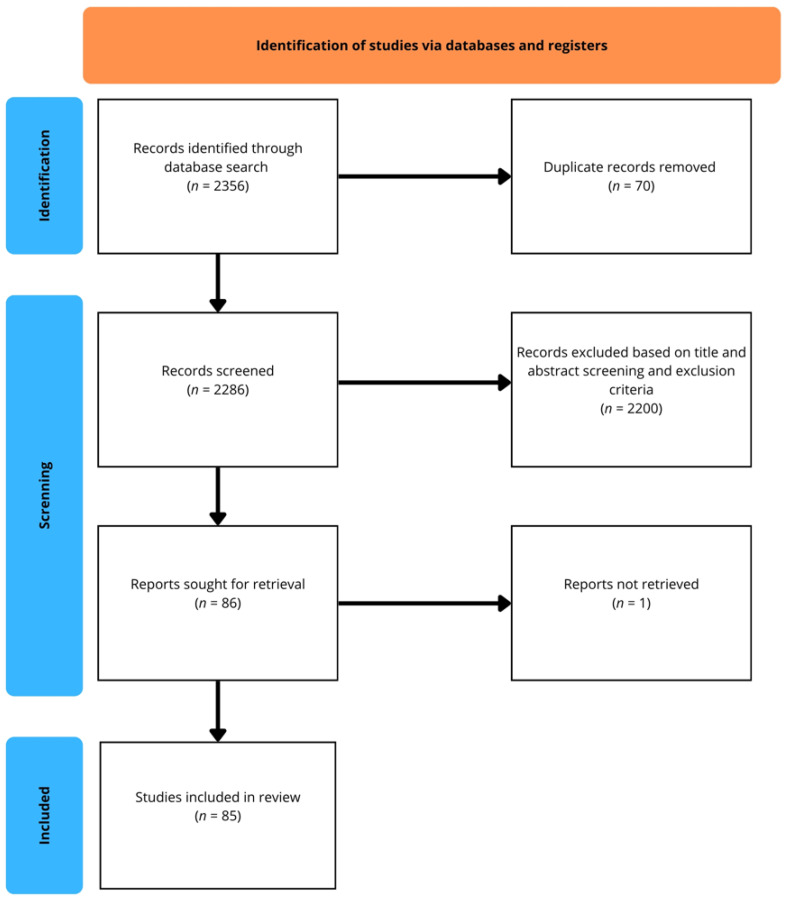
Flow diagram of the study selection process.

A structured literature search was conducted across multiple databases (PubMed/MEDLINE, EMBASE, Scopus, Cochrane Library, Sage Journals, ScienceDirect, Taylor & Francis Online, and SpringerLink). The initial search yielded a total of 2356 records. After the removal of 70 duplicates, 2286 records were screened based on titles and abstracts. Of these, 2200 were excluded for not meeting the predefined eligibility criteria. A total of 86 reports were sought for full-text retrieval; however, one could not be accessed. Finally, 85 studies met the inclusion criteria and were incorporated into the qualitative synthesis. The selection process is illustrated in the flow diagram (Figure 1).

### 3.9. Methodological Quality

#### 3.9.1. Evaluation Using the JBI Critical Appraisal Tool

In Section 3.9.1, two reviewers (J.C.F. and E.Z.M.) applied the JBI Critical Appraisal Tool for Systematic Reviews, Textual Evidence: Narrative, Cohort Studies, Analytical Cross-Sectional Studies, Case Series, Case–Control Studies. Details of these assessments are presented in Table 1, Table 2, Table 3, Table 4, Table 5 and Table 6.

#### 3.9.2. Summary of Evidence Quality

Table 7 shows that β-lactams and vancomycin reach B-level evidence with broadly consistent ECMO findings (circuit effects modest; renal function/CRRT drives variability), supporting extended/continuous infusions and TDM. Antifungals and sedatives are mostly C–D, with under-exposure (e.g., voriconazole) or early sequestration (e.g., midazolam/propofol), reinforcing trough or AUC-guided approaches and individualized dosing. Formal systematic reviews do exist, but their own conclusions are that adult ECMO data are sparse/heterogeneous and often observational, with few drug-specific, consistent dosing conclusions so they do not lift any single drug to Grade A. In contrast, several agents do reach Grade B thanks to prospective multicenter studies that analyses population pharmacokinetics or comparative cohorts (e.g., vancomycin, piperacillin–tazobactam, meropenem, amikacin, UFH monitoring strategies), but not A.

### 3.10. Drugs with No ECMO Data

Using our predefined search strategy and inclusion/exclusion criteria, we did not identify any adult ECMO-specific pharmacokinetic or pharmacodynamic data for several clinically relevant agents. These included the non-opioid analgesics metamizole (dipyrone) and paracetamol (acetaminophen); the local anesthetic lidocaine; the antimicrobial agents metronidazole, ganciclovir, and acyclovir; the neuromuscular blocking agents atracurium and pancuronium; and parenteral electrolyte supplements (e.g., potassium chloride, magnesium sulfate, calcium gluconate, phosphate).

### 3.11. Guideline with Suggest Recommendations

To facilitate clinical application, the evidence synthesized in this review was organized into a structured pharmacotherapy guide (Table 8). The table provides a concise summary of key pharmacotherapeutic aspects for medications commonly used in critically ill adult ECMO patients, grouped by therapeutic class. For each drug, essential information is presented, including dosage recommendations, pharmacokinetic/pharmacodynamic considerations, therapeutic drug monitoring targets, available alternatives, and clinical indications or precautions. This format allows rapid comparison across drug classes and highlights areas where robust evidence exists versus those where data remain scarce. Given the high PK variability in ECMO, embedding MIPD/TDM for time-dependent drugs aligns with randomized data showing improved feasibility of target-attainment strategies in the general ICU, and is likely even more relevant in ECMO patients [37]. Unless explicitly noted as ECMO practice–specific are derived from general ICU literature and applied to ECMO, for which adult studies confirm high variability but do not define new thresholds.

## 4. Discussion

**Figure 2:**
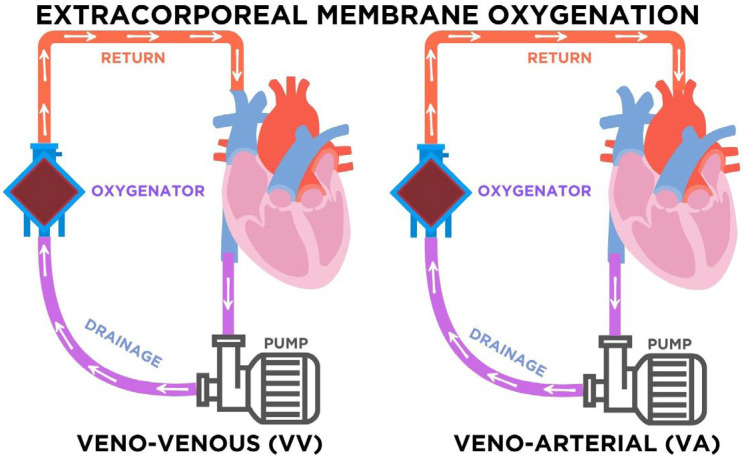
Configurations of extracorporeal membrane oxygenation (ECMO). In veno-venous (VV) ECMO, venous blood is drained, pumped through the oxygenator, and returned to the venous system, providing respiratory support. In veno-arterial (VA) ECMO, venous blood is drained and returned to the arterial system, supporting both respiratory and circulatory function. Arrows indicate the direction of blood flow through the circuit. The purple arrows represent deoxygenated blood draining from the venous system toward the pump and oxygenator, while the red arrows represent oxygenated blood returning to the patient. The blue components correspond to the oxygenator, where gas exchange occurs.

The use of extracorporeal membrane oxygenation (ECMO) has transitioned from a last-resort salvage therapy to a widely applied intervention in intensive care units [1,2,3,4]. As illustrated in Figure 2, ECMO consists of a centrifugal pump and a membrane oxygenator that drains venous blood, removes carbon dioxide, and returns oxygenated blood to the patient’s circulation. In veno-venous (VV) ECMO, blood is drained and reinfused into the venous system, providing exclusive respiratory support while preserving native cardiac output. In contrast, veno-arterial (VA) ECMO reinfuses blood into arterial circulation, supporting both respiratory and circulatory function. The choice of modality depends on the clinical condition: VV-ECMO is preferred in severe isolated respiratory failure such as ARDS, whereas VA-ECMO is indicated in refractory cardiogenic shock or profound cardiac dysfunction [1,2,3,4]. This distinction is relevant not only physiologically but also pharmacologically, since modality-specific factors influence drug distribution, anticoagulation needs, and vasopressor requirements [61,64,84,87,96].

**Figure 3:**
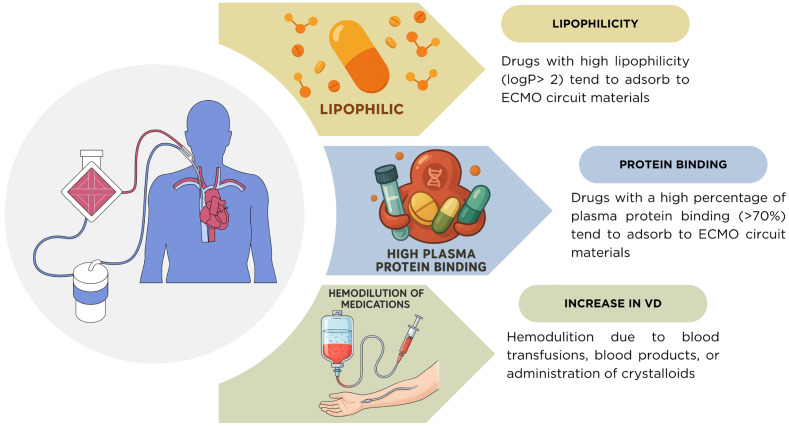
Mechanisms of pharmacokinetic alterations during ECMO. Drugs with high lipophilicity (logP > 2) and high plasma protein binding (>70%) tend to adsorb onto ECMO circuit components, reducing plasma concentrations. In addition, the volume of distribution (Vd) is frequently increased due to hemodilution from blood transfusions, blood products, or crystalloid administration, further complicating drug exposure in critically ill ECMO patients.

Another fundamental determinant of pharmacokinetic (PK) variability during ECMO is the interaction between drug properties and fluid dynamics. As shown in Figure 3 and Table 9, drugs with high lipophilicity (logP > 2) and plasma protein binding greater than 70% are highly susceptible to adsorption within ECMO circuit components, leading to subtherapeutic plasma concentrations [5,22]. In addition, critically ill patients frequently undergo hemodilution from blood transfusions, administration of blood products, and crystalloid infusion used to maintain circuit flow. These interventions expand the apparent volume of distribution (Vd) and contribute to underexposure, compounding the impact of circuit adsorption [6,7]. The combination of these factors underscores the need for individualized dosing and therapeutic drug monitoring (TDM) in ECMO patients.

**Figure 4:**
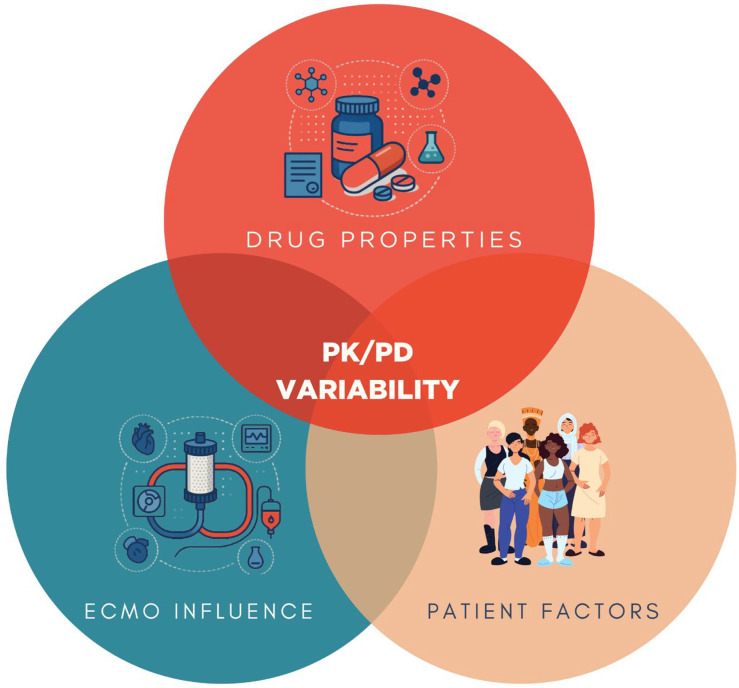
Conceptual framework of PK/PD variability in ECMO. Drug properties (lipophilicity, protein binding, molecular weight, volume of distribution), patient factors (opioid tolerance, body mass index, fluid balance, hepatic and renal function), and ECMO circuit influence (drug sequestration by circuit components) interact to determine pharmacokinetic and pharmacodynamic alterations in critically ill patients supported with ECMO.

Pharmacokinetic and pharmacodynamic (PK/PD) variability in ECMO is ultimately the result of a triad of influences: drug-related, patient-specific, and ECMO circuit-related factors. As illustrated in Figure 4, the properties of each medication including lipophilicity, protein binding, molecular weight, and volume of distribution serve as primary determinants of sequestration and clearance [5,22]. Patient-related factors such as opioid tolerance, body mass index, fluid balance, hepatic and renal function, and systemic inflammation further modulate drug disposition and effect [6,7]. In parallel, the ECMO circuit itself adsorbs lipophilic and protein-bound drugs onto oxygenator membranes and tubing surfaces [5,22]. This interplay explains much of the interindividual variability observed in drug exposure and reinforces the need for precision pharmacotherapy based on mechanistic understanding, clinical context, and TDM.

It is also important to situate adult findings within the broader ECMO literature. Pharmacokinetic alterations during ECMO are not homogeneous across age groups. Neonatal and infant studies have demonstrated distinct maturational clearance and circuit/device effects, underscoring the strong influence of developmental physiology on drug disposition [8]. These age-dependent differences caution against extrapolating pediatric or neonatal data to adults. By explicitly separating adult evidence, the present review highlights that dosing strategies must be tailored to age group and clinical context, and that robust adult-specific pharmacokinetic data remains essential for optimizing therapy.

Clinical data consistently show that opioids and sedatives behave differently during ECMO. Morphine, due to its hydrophilic nature and moderate protein binding, demonstrates limited sequestration; however, accumulation of active metabolites in renal dysfunction requires close monitoring [11,12,13,14]. In contrast, fentanyl is highly lipophilic and protein-bound, making it prone to unpredictable adsorption and increased dose requirements [15,16,17,18]. Benzodiazepines, particularly midazolam, accumulate significantly, leading to delayed awakening and increased risk of delirium [22,24]. Propofol, while susceptible to circuit adsorption, generally achieves sedation targets with careful titration, though clinicians must remain vigilant for metabolic complications such as hypertriglyceridemia and propofol infusion syndrome [15,23]. Ketamine appears less affected by ECMO circuits, with observed variability largely attributable to the underlying critical illness rather than the extracorporeal system itself [19,20,21].

Infection management in ECMO patients represents one of the most consequential pharmacotherapeutic challenges. Hydrophilic antibiotics such as β-lactams (cefotaxime, ceftazidime, meropenem, piperacillin–tazobactam) and aminoglycosides (amikacin) generally exhibit minimal circuit sequestration, but altered distribution volumes and clearance frequently necessitate prolonged or continuous infusion strategies to maintain time-dependent exposure [29,30,31,32,33,34,35,36,48,79,90,91,92,93,98,108,109,113,114]. Vancomycin is minimally influenced by the circuit itself; however, high interpatient variability driven by renal function and systemic inflammation underscores the importance of AUC-guided TDM [53,54,55,56,57,58,59,60,99]. Linezolid demonstrates inconsistent plasma concentrations, with frequent subtherapeutic exposures, highlighting the need for individualized monitoring [49,50]. Aminoglycosides, although not heavily sequestered, often require higher loading doses to achieve bactericidal peaks [29,30,31,32,33].

Antifungal therapy illustrates the heterogeneity of ECMO pharmacokinetics. Fluconazole remains reassuringly stable [39,83]. By contrast, voriconazole and posaconazole frequently show subtherapeutic concentrations despite standard dosing, mandating dose escalation and rigorous TDM [44,45,46,47]. Caspofungin appears relatively unaffected, though higher doses may be required in patients with elevated body weight or severe illness [81,82,94,95]. Liposomal amphotericin B is particularly problematic, with pronounced circuit adsorption leading to highly variable plasma concentrations and unpredictable efficacy [39,40,43].

Evidence for antiviral therapy in ECMO is almost entirely lacking. Drugs such as ganciclovir and acyclovir have no ECMO-specific PK/PD data, and dosing recommendations are currently extrapolated from non-ECMO populations. This represents a critical evidence gap that urgently requires targeted investigation.

Vasopressor and inotrope use in ECMO highlights the interplay between illness severity and pharmacokinetics. Norepinephrine remains the most widely used first-line vasopressor, with minimal ECMO-specific alterations; observed dose variability largely reflects disease severity [61,62,63,84]. Epinephrine use in VA-ECMO, however, has been associated with increased mortality and adverse outcomes, and should therefore be reserved for refractory situations [64,65]. Dobutamine data are limited, but high-dose use carries risks of arrhythmias and cardiomyopathy; alternatives such as milrinone or levosimendan are generally favored [66].

Anticoagulation is central to ECMO management but fraught with variability. Unfractionated heparin remains the global standard; however, interpatient differences in inflammation, antithrombin activity, and organ function make dose adjustment highly individualized. Anti-FXa monitoring provides more consistent results than ACT or aPTT, though multimodal strategies are increasingly recommended [67,68,69,70]. Enoxaparin is an emerging alternative with a more predictable PK profile, though clinical evidence is limited [73,74]. Direct thrombin inhibitors such as bivalirudin and argatroban are attractive options due to more predictable PK, but their broader adoption remains limited by cost and lack of multicenter outcome data [75,76,89,96].

The data about neuromuscular blockers is scarce, rocuronium, being lipophilic and hepatically metabolized, may accumulate during ECMO, raising concerns about prolonged neuromuscular blockade [22,77,78]. By contrast, cisatracurium metabolized independently of hepatic or renal function remains the agent of choice due to its more predictable pharmacokinetics [22,77,78].

In commonly used electrolytes such as calcium gluconate, magnesium sulfate, potassium chloride, and potassium phosphate, no ECMO-specific PK data exist. Current dosing is extrapolated from conventional ICU practice, with therapy guided by serial serum monitoring and patient-specific requirements. These gaps represent an underexplored yet clinically important area of ECMO pharmacotherapy.

In summary, physicochemical drug properties provide important but incomplete predictors of ECMO-related PK/PD alterations, as patient physiology and circuit factors exert substantial influence. TDM is indispensable for high-risk medications such as vancomycin, aminoglycosides, linezolid, and azole antifungals. Extended or continuous infusion strategies should be prioritized for β-lactams. Clinicians should favor drugs with more predictable PK behavior when alternatives exist (e.g., morphine over fentanyl, cisatracurium over rocuronium). However, current evidence remains limited by small, retrospective, and single-center studies, underscoring the urgent need for multicenter trials and standardized monitoring protocols.

## 5. Conclusions

Optimizing pharmacotherapy in ECMO-supported critically ill adults requires integrating drug physicochemical properties, patient-specific physiology, and circuit-related influences into individualized treatment strategies. Lipophilic and highly protein-bound medications are most vulnerable to sequestration, while hydrophilic agents are affected predominantly by organ dysfunction and fluid shifts. Therapeutic drug monitoring (TDM) remains essential for high-risk drugs, including vancomycin, aminoglycosides, linezolid, and azole antifungals, whereas prolonged or continuous infusions should be prioritized for β-lactams. When possible, clinicians should favor medications with predictable PK/PD behavior, such as morphine or cisatracurium, over agents with high variability. Current evidence is constrained by small, retrospective, or ex vivo studies, with limited modality-specific (VV vs. VA) comparisons. Future priorities include multicenter clinical trials, standardized TDM protocols, and physiologically based PK modeling to refine dosing recommendations across drug classes. Bridging these knowledge gaps will be crucial to ensure safer and more effective pharmacotherapy in patients requiring ECMO support.

## 6. Future Directions

Several clinically relevant drugs remain without adult ECMO-specific PK/PD data, including paracetamol, metamizole, lidocaine, metronidazole, acyclovir, ganciclovir, atracurium, pancuronium, and commonly administered electrolytes. Future work should prioritize prospective population PK studies in these domains. For neuromuscular blocking agents, research is needed to clarify circuit adsorption and define depth-of-block monitoring strategies. For antivirals such as acyclovir and ganciclovir, studies should integrate PK with viral load dynamics, renal replacement therapy, and circuit covariates. For widely used non-opioid analgesics and antibiotics, prospective PK/PD investigations are required to determine whether ECMO alters distribution or clearance. Finally, systematic kinetic studies of intravenous electrolytes (potassium, magnesium, calcium, phosphate) could inform pragmatic replacement algorithms tailored to ECMO patients. Addressing these knowledge gaps will be critical to reduce reliance on extrapolation from non-ECMO ICU cohorts and to build a more comprehensive evidence base for drug dosing in adult ECMO.

## Figures and Tables

**Table 1:** Methodological Quality Assessment of Prevalence Studies Using the JBI Critical Appraisal Tool for Systematic Reviews.

Item	Review Question Clearly and Explicitly	Appropriate Inclusion Criteria	Appropriate Search Strategy	Adequate Sources and Resources	Appropriate Criteria for Appraising Studies	≥2 Reviewers	Methods to Minimize Errors in Data Extraction	Appropriate Methods Used to Combine Studies	Likelihood of Publication Bias Assessed	Recommendations for Policy and/or Practice Supported by the Reported Data	Appropriate Specific Directives for New Research
Flanagan et al., 2024 [19]	Yes	Yes	Yes	Yes	Yes	Yes	Yes	Yes	Yes	Yes	Yes
Duceppe et al., 2021 [33]	Yes	Yes	Yes	Yes	Yes	Yes	Yes	Yes	Unclear	Yes	Yes
Jendoubi et al., 2024 [39]	Yes	Yes	Yes	Yes	Yes	Yes	Yes	Yes	Unclear	Yes	Yes
Zhao et al., 2024 [66]	Yes	Yes	Yes	Yes	Yes	Yes	Yes	Yes	Unclear	Yes	Yes
Willems et al., 2021 [70]	Yes	Yes	Yes	Yes	Yes	Yes	Yes	Yes	Unclear	Unclear	Yes

**Table 2:** Methodological Quality Assessment of Prevalence Studies Using the JBI Critical Appraisal Tool for Narrative Reviews.

Item	Credible or Appropriate Source	Relationship Between the Text and Its Context Explained	Logical Sequence	Arrive at Similar Conclusions to Those Drawn by the Narrator	The Conclusions Flow From the Narrative	This Account to Be a Narrative
Patel 2023 [5]	Yes	Yes	Yes	Yes	Yes	Yes
Ochroch 2021 [11]	Yes	Yes	Yes	Yes	Yes	Yes
Shah 2017 [13]	Yes	Yes	Yes	Yes	Yes	Yes
Meng 2017 [14]	Yes	Yes	Yes	Yes	Yes	Yes
Dzierba 2023 [15]	Yes	Yes	Yes	Yes	Yes	Yes
Dreucean 2022 [22]	Yes	Yes	Yes	Yes	Yes	Yes
Ha & Sieg 2017 [24]	Yes	Yes	Yes	Yes	Yes	Yes
Sulaiman 2022 [38]	Yes	Yes	Yes	Yes	Yes	Yes
Lyster 2023 [40]	Yes	Yes	Yes	Yes	Yes	Yes
Tanaka 2025 [50]	Yes	Yes	Yes	Yes	Yes	Yes
Hahn 2017 [53]	Yes	Yes	Yes	Yes	Yes	Yes
Vajter & Volod 2025 [67]	Yes	Yes	Yes	Yes	Yes	Yes
Pollak 2019 [76]	Yes	Yes	Yes	Yes	Yes	Yes
Bakdach 2022 [79]	Yes	Yes	Yes	Yes	Yes	Yes
Roberts 2022 [80]	Yes	Yes	Yes	Yes	Yes	Yes
Albanell-Fernández 2025 [81]	Yes	Yes	Yes	Yes	Yes	Yes
Peitz & Murry 2023 [82]	Yes	Yes	Yes	Yes	Yes	Yes
Bellmann 2017 [83]	Yes	Yes	Yes	Yes	Yes	Yes
Vo 2025 [84]	Yes	Yes	Yes	Yes	Yes	Yes

**Table 3:** Methodological Quality Assessment of Prevalence Studies Using the JBI Critical Appraisal Tool for Cohort Studies.

Item	Two Groups Similar and Recruited from the Same Population	Measured Similarly to Assign People to Both Groups	Exposure Measured in a Valid and Reliable Way	Identified Confounding Factors	Strategies to Deal with Confounding Factors	Groups/Participants Free of the Outcome at the Start of the Study	Measured Outcomes in a Valid and Reliable Way	Follow Up Time Reported and Sufficient to Be Long Enough for Outcomes	Follow Up Complete, and if not, Were the Reasons to Loss to Follow Up Described and Explored	Strategies to Address Incomplete Follow Up	Appropriate Statistical Analysis
Nigoghossian 2016 [16]	Yes	Yes	Yes	Yes	Yes	Yes	Yes	Yes	Yes	NA	Yes
Landolf 2020 [18]	Yes	Yes	Yes	Yes	Yes	Yes	Yes	Yes	Yes	NA	Yes
Lamm 2019 [23]	Yes	Yes	Yes	Yes	Yes	Yes	Yes	Yes	Yes	NA	Yes
Schaller 2025 [28]	Yes	Yes	Yes	Yes	Yes	Yes	Yes	Yes	Yes	NA	Yes
Cheng 2022 [55]	Yes	Yes	Yes	Yes	Yes	Yes	Yes	Yes	Yes	NA	Yes
Ferre 2024 [56]	Yes	Yes	Yes	Yes	Yes	Yes	Yes	Yes	Yes	NA	Yes
Park 2015 [57]	Yes	Yes	Yes	Yes	Yes	Yes	Yes	Yes	Yes	NA	Yes
Jung 2021 [58]	Yes	Yes	Yes	Yes	Yes	Yes	Yes	Yes	Yes	NA	Yes
Distelmaier 2020 [61]	Yes	Yes	Yes	Yes	Yes	Yes	Yes	Yes	Yes	NA	Yes
Jacky 2018 [63]	Yes	Yes	Yes	Unclear	Yes	Yes	Yes	Yes	Yes	NA	Yes
Massart 2021 [65]	Yes	Yes	Yes	Yes	Yes	Yes	Yes	Yes	Yes	NA	Yes
Feng 2024 [68]	Yes	Yes	Yes	Unclear	Yes	Yes	Yes	Yes	Yes	NA	Yes
Robinson 2025 [71]	Yes	Yes	Unclear	Unclear	Yes	Yes	Yes	Yes	Yes	NA	Yes
Wiegele 2022 [73]	Yes	Yes	Yes	Yes	Yes	Yes	Yes	Yes	Yes	NA	Yes
Diaz 2023 [75]	Yes	Yes	Yes	Unclear	Yes	Yes	Yes	Yes	Yes	NA	Yes
Browder 2022 [85]	Yes	Yes	Yes	Unclear	Yes	Yes	Yes	Yes	Yes	NA	Yes
Gratz 2020 [86]	Yes	Yes	Yes	Unclear	Yes	Yes	Unclear	Yes	Yes	NA	Yes
Seelhammer 2021 [87]	Yes	Yes	Yes	Yes	Yes	Yes	Yes	Yes	Yes	NA	Yes
Barker 2020 [88]	Yes	Yes	Yes	Yes	Yes	Yes	Yes	Yes	Yes	NA	Yes
Taha 2025 [89]	Yes	Yes	Yes	Unclear	Yes	Yes	Yes	Yes	Yes	NA	Yes

**Table 4:** Methodological Quality Assessment of Prevalence Studies Using the JBI Critical Appraisal Tool for Analytical Cross-Sectional Studies.

Item	Clearly Defined Criteria for Inclusion	Study Subjects and the Setting Described in Detail	Exposure Measured in a Valid and Reliable Way	Objective, Standard Criteria Used for Measurement of the Condition	Identified Confounding Factors	Stated Strategies to Deal with Confounding Factors	Outcomes Measured in a Valid and Reliable Way	Appropriate Statistical Analysis
Ren 2021 [12]	Yes	Yes	Yes	Yes	Yes	Yes	Yes	Yes
Patel 2020 [17]	Yes	Yes	Yes	Yes	Yes	Yes	Yes	Yes
Touchard 2018 [29]	Yes	Yes	Yes	Yes	Yes	Yes	Yes	Yes
Bouglé 2019 [30]	Yes	Yes	Yes	Yes	Yes	Yes	Yes	Yes
Ruiz-Ramos 2018 [31]	Yes	Yes	Yes	Yes	Yes	Yes	Yes	Yes
Pressiat 2022 [32]	Yes	Yes	Yes	Yes	Yes	Yes	Yes	Yes
Hanberg 2018 [34]	Yes	Yes	Yes	Yes	Yes	Yes	Yes	Yes
Kang 2022 [35]	Yes	Yes	Yes	Yes	Yes	Yes	Yes	Yes
Gijsen 2021 [36]	Yes	Yes	Yes	Yes	Yes	Yes	Yes	Yes
Shekar 2023 [48]	Yes	Yes	Yes	Yes	Yes	Yes	Yes	Yes
Liu 2020 [54]	Yes	Yes	Yes	Yes	Yes	Yes	Yes	Yes
Lanoiselée 2024 [72]	Yes	Yes	Yes	Yes	Yes	Yes	Yes	Yes
Durila 2025 [74]	Yes	Yes	Yes	Yes	Yes	Yes	Yes	Yes
Curtiaud 2024 [90]	Yes	Yes	Yes	Yes	Yes	Yes	Yes	Yes
Cheng 2021 [91]	Yes	Yes	Yes	Yes	Yes	Yes	Yes	Yes
Hahn 2021 [92]	Yes	Yes	Yes	Yes	Yes	Yes	Yes	Yes
Kim 2022 [93]	Yes	Yes	Yes	Yes	Yes	Yes	Yes	Yes
Abdul-Aziz 2025 [94]	Yes	Yes	Yes	Yes	Yes	Yes	Yes	Yes
Wang 2020 [95]	Yes	Yes	Yes	Yes	Yes	Yes	Yes	Yes
Cartwright 2021 [96]	Yes	Yes	Yes	Yes	Yes	NA	Yes	Yes

**Table 5:** Methodological Quality Assessment of Prevalence Studies Using the JBI Critical Appraisal Tool for Case Series.

Item	Clear Inclusion Criteria	Condition Measured in a Standard, Reliable Way for All Participants	Valid Methods Used for Identification of the Condition for all Participants	Consecutive Inclusion of Participants	Complete Inclusion of Participants	Clear Reporting of the Demographics of the Participants	Clear Reporting of Clinical Information of the Participants	Clearly Reported Outcomes or Follow Up Results	Clear Reporting of the Presenting Site(s)/Clinic(s) Demographic Information	Appropriate Statistical Analysis
Timofte 2017 [25]	Yes	Yes	Yes	Unclear	Unclear	Yes	Yes	Yes	Yes	NA
Torbic 2022 [62]	Yes	Yes	Yes	Yes	Yes	Yes	Yes	Yes	Yes	NA
Wicky 2023 [97]	Yes	Yes	Yes	Yes	Yes	Yes	Yes	Yes	Yes	Yes

**Table 6:** Methodological Quality Assessment of Prevalence Studies Using the JBI Critical Appraisal Tool for Case–Control Studies.

Item	Comparable Groups	Cases and Controls Matched Appropriately	Same Criteria Used for Identification of Cases and Controls	Exposure Measured in a Standard, Valid and Reliable Way	Exposure Measured in the Same Way for Cases and Controls	Identified Confounding Factors	Stated Strategies to Deal with Confounding Factors	Outcomes Assessed in a Standard, Valid and Reliable Way for Cases and Controls	Exposure Period of Interest Long Enough to Be Meaningful	Appropriate Statistical Analysis
Ronda 2023 [46]	Yes	Unclear	Yes	Yes	Yes	Yes	Yes	Yes	Yes	Yes
Donadello 2015 [98]	Yes	Yes	Yes	Yes	Yes	Yes	Yes	Yes	Yes	Yes
Wu 2016 [99]	Yes	Yes	Yes	Yes	Yes	Yes	Yes	Yes	Yes	Yes

**Table 7:** Evidence grading and standardized PK/PD targets for commonly used drugs during ECMO.

Drug	Standardized PK/PD Target	Evidence Grade
Morphine	Clinical effect (RASS)	C
I Fentanyl	Clinical effect (RASS)	C
Ketamine	Clinical effect (RASS)	D
Propofol	Clinical effect (RASS)	C–D
Diazepam	Clinical effect (RASS)	D
Midazolam	Clinical effect (RASS)	C
Amikacin	Cmax/MIC 8–10 (practical Cmax ≥ 60–80 mg/L, Cmin < 5 mg/L)	B
Cefotaxime	%fT > MIC 50–100%	C–D
Ceftazidime	%fT > MIC 100% (CI 6 g/day when needed)	C
Meropenem	%fT > MIC 100% (or 100% fT > 4 × MIC severe); prefer extended/continuous	B
Ertapenem	%fT > MIC ≥ 50–100%	D
Linezolid	Cmin 2–7 mg/L (AUC/MIC ≥80)	C
Vancomycin	AUC24/MIC 400–600 (AUC-guided preferred)	B
Piperacillin–Tazobactam	%fT > MIC ≥ 50–100% (or %fT > 4 × MIC severe); favor 4 h or continuous infusions	B
Tigecycline	AUC/MIC (no universal bedside threshold)	C
Amphotericin B	Clinical response/safety (no validated PK target)	C–D
Liposomal Amphotericin B	Clinical response/safety	C
Caspofungin	AUC/MIC (no validated clinical cut-off)	B-
Fluconazole	Cmin 10–15 mg/L (or AUC/MIC ≥100)	B-
Voriconazole	Cmin 2–5 mg/L	B-
Epinephrine	Clinical hemodynamic goals	B
Dobutamine	Clinical hemodynamic goals	C–D
Norepinephrine	Clinical hemodynamic goals	C
Unfractionated Heparin (UFH)	Anti-Xa 0.3–0.7 IU/mL (preferred), aPTT 50–90 s, ACT 180–220 s	B
Enoxaparin (SC or IV CI)	SC: peak anti-Xa 0.3–0.5 IU/mL; IV CI: anti-Xa 0.4–0.6 IU/mL	C/C+
Rocuronium	Clinical neuromuscular depth (TOF/EMG)	C–D

Abbreviations: Richmond Agitation-Sedation Scale (RASS).

**Table 8:** Management and Considerations of Commonly used medications in the Intensive Care Unit (ICU) in patients undergoing ECMO therapy.

INN (Drug)	Dosage Recommendations	PK/PD Considerations	Therapeutic Drug Monitoring	Alternatives (If Needed)	Clinical Indications & Precautions
Morphine	-IV Bolus: 2–4 mg every 1–2 h -Continuous infusion: 2–30 mg/h, titrated according to clinical response and TDM [13,100].	-Moderate protein binding and low lipophilicity making morphine less susceptible to ECMO-related sequestration compared to other opioids [5,14]. -ECMO has minimal circuit effect; Vd/CL/AUC/t½ mostly reflect organ dysfunction in critical illness [13,14,22].	-Monitor using CPOT < 3 or BPS < 5 and light sedation (RASS −2 to 0); targets from general ICU practice, not ECMO-specific [14]. -Adjust for renal/hepatic dysfunction and clinical response [12,13,22].	Ketamine, dexmedetomidine, IV acetaminophen, IV lidocaine [11].	-Commonly used for analgesia and sedation as part of multimodal therapy [11]. -Histamine release may cause profound hypotension; caution in hemodynamically unstable patients [5].
Fentanyl	-Intermittent IV Bolus: 0.35–0.5 μg/kg every 30–60 min [13]. -Continuous infusion: 0.7–10 μg/kg/h (typically initiated at 25–100 μg/h [17,101]. -Possibly dose escalation ≥2000 μg/day often required due to ECMO sequestration [16].	ECMO adsorption early → ↑ apparent Vd, variable AUC; later accumulation driven by organ dysfunction, subsequent variability mainly from critical illness and organ failure [5,15].	CPOT < 3, BPS < 5 and RASS −2 to 0; thresholds from general ICU practice; reassess daily for opioid-sparing strategies and de-escalation [15,22].	Consider less lipophilic opioids like hydromorphone or analgesic adjuncts such as ketamine, dexmedetomidine, IV lidocaine, or IV acetaminophen [5,15,18,85].	Requires careful dose titration to balance analgesic efficacy with risks of sedation, opioid tolerance, and side effects [17].
Ketamine	-IV bolus: 0.1–0.5 mg/kg (up to 1–4.5 mg/kg for severe agitation) [5,13,19,21]. -Continuous infusion: 0.05–2.5 mg/kg/h (commonly 0.5–2 mg/kg/h), titrated to achieve defined sedation targets (e.g., RASS) [5,13,15,19,22].	-Moderate lipophilicity, low protein binding, and increased volume of distribution suggest possible ECMO-related sequestration, but significant clinical impact unlikely [5,19,20,102]. -Limited ECMO data, ↑ apparent Vd; dosing variability and Vd/CL changes more related to critical illness than ECMO-specific effects [5,19,20,102].	Titrate to RASS −2 to 0; general ICU target; no ECMO-specific numeric PK goals; monitor for psychomimetic effects and hemodynamic changes [13,15,22].	Dexmedetomidine, opioids, IV acetaminophen, or lidocaine as adjuncts within a multimodal analgesia sedation strategy [5,19].	-Recommended as part of a multimodal therapy [5,13,15,19,22]. -Higher initial doses or continuous infusion may be required in ECMO patients, with special attention in critically ill patients due to increased volume of distribution and potential ECMO sequestration [5,13,15,19,22].
Propofol	-IV bolus: 5 μg/kg/min IV for 5 min [13]. -Continuous infusion: 5–50 μg/kg/min IV, titrated based on clinical sedation targets (RASS: typically −1 to −2) [12,13]. -Avoid prolonged high-dose infusion (>50 μg/kg/min) to minimize risks such as PRIS and hypertriglyceridemia [22].	-High lipophilicity and extensive protein binding result in significant adsorption, especially notable early during ECMO therapy [14,22,103,104]. -ECMO adsorption early → ↑ Vd; CL/AUC affected by hemodynamics/organ failure; t½ may lengthen with prolonged use [14,22,103,104]. -Despite sequestration, minimal impact observed clinically on ECMO oxygenator life and no significant changes reported in triglycerides, free hemoglobin, fibrinogen, or platelet levels even with prolonged use [23].	-Target RASS −2 to 0; check serum triglycerides at baseline and every 48–72 h (every 24–48 h if >4 mg/kg/h or >72 h infusion); discontinue if >400–500 mg/dL or signs of propofol infusion syndrome; general ICU thresholds, daily reassessment for de-escalation [22]. -Individual dose titration critical to optimize therapeutic effect while minimizing adverse outcomes [13,14].	Midazolam, dexmedetomidine, ketamine, or opioids like hydromorphone as adjuncts or alternatives within multimodal sedation strategies, especially if high propofol doses become necessary or clinically problematic [85].	-Preferred agent for short-term sedation in critically ill ECMO patients. Caution due to potential negative inotropic and chronotropic effects [17]. -Carefully titrate according to sedation and hemodynamic goals, ECMO sequestration often necessitates higher than typical ICU doses [22,103,104].
Diazepam	-IV intermittent bolus: 5–10 mg administered 15–30 min before neuromuscular blockade [25]. -Total daily doses ranging from 170 to 260 mg/day reported for agitation control, amnesia, and hemodynamic optimization [25]. -Routine or prolonged use not recommended in ECMO patients [22].	High PB + lipophilicity → ECMO adsorption/↑ Vd and prolonged t½; CL limited by hepatic function → accumulation, pose significant risk for accumulation and sequestration in ECMO circuits [22].	Aim RASS −2 to −3; monitor for delayed awakening/accumulation; general ICU bedside targets; consider switch to shorter-acting agents if excessive effect observed [22,25].	Prefer alternative agents with lower sequestration risk and shorter half-life, such as midazolam, dexmedetomidine [22].	-Diazepam’s use in ECMO is generally discouraged due to pharmacokinetic properties that significantly increase accumulation risk [22,25]. -Use cautiously in short-term situations requiring rapid sedation or amnesia before neuromuscular blockade, closely monitoring for sedation-related complications [22,25].
Midazolam	-IV loading dose: 0.01–0.05 mg/kg. -Continuous infusion: 1–7 mg/h (0.02–0.1 mg/kg/h, adjusted by weight), titrated based on clinical sedation targets (RASS) [5,13]. -Reported median daily dose of 280 mg/day (IQR: 209–384 mg/day) or equivalent during periods of deep sedation in ECMO patients [12,16].	-ECMO-specific early sequestration; critical-illness factors (hepatic dysfunction, accumulation) dominate later PK. [5,22]. -ECMO adsorption early → ↑ Vd; later accumulation (active metabolites) if hepatic dysfunction; AUC ↑, t½ ↑; CL ↓ variably. -Potential accumulation of active metabolites, especially in hepatic dysfunction [22,24].	Aim for RASS −2 to −3; general ICU targets; monitor for accumulation (delayed awakening, prolonged ventilation, burst suppression if EEG available), along with regular assessment of hepatic and renal function [5,13,105].	Dexmedetomidine, ketamine, or non-benzodiazepine sedatives recommended as alternatives, particularly in hepatic impairment or when metabolite accumulation is a concern [22,24].	-Widely used for deep sedation in ECMO-supported patients, but dosing requirements are highly variable due to ECMO circuit sequestration and critical illness-related pharmacokinetics [12,13,16]. -Individualized TDM essential to optimize sedation and minimize adverse effects [12,13,16].
Amikacin	-Standard dosing: 25–30 mg/kg IV as a single daily dose infused over 30 min [13,29,33,106]. -Renal function based adjustments: KDIGO 0 (normal renal function): up to 40 mg/kg IV. KDIGO 3 (severe acute kidney injury): 25 mg/kg IV [32]. -BMI and fluid balance-based adjustments: BMI ≥ 22 kg/m^2^ with negative fluid balance: 25 mg/kg. BMI ≥ 22 kg/m^2^ with positive fluid balance: ≥30 mg/kg [29]. -Maintenance dose (if trough <5 mg/L at 24 h): 15–20 mg/kg IV every 24 h [106,107].	Hydrophilic antibiotic, low protein binding. ECMO effect negligible; ↑ Vd from fluid shifts → low peaks; CL driven by ARC/AKI; AUC variable; t½ depends on renal function [5,33].	Recommended therapeutic drug monitoring (TDM) with target plasma levels: Cmax/MIC 8–10 (Cmax 60–80 mg/L; Cmin < 5 mg/L at 24 h); general ICU PK/PD target; ECMO studies confirm underexposure but keep same goals. Monitor closely due to significant variability from fluid balance, renal function, patient weight, and severity of critical illness [13,29,30,31,32].	*	Primary indication: severe MDR Gram-negative bacterial infections. ECMO does not independently necessitate dose adjustment, but dosing variability is high due to patient-specific factors [29,30,31,32,33].
Cefotaxime	-Standard daily dose: Standard dosing: 2 g IV q6–8 h over 30 min. Extended infusion (3–4 h) can be used in severe infection [30]. -Median effective daily dose reported: 7 g/day (IQR: 6–8 g/day), effectively achieving therapeutic plasma concentrations (CT50 ≥ 4 mg/L, Cmin ≥1 mg/L) without requiring ECMO-specific adjustments [30].	-β-lactam antibiotic with time-dependent efficacy (CT50 ≥ 4 mg/L and Cmin ≥ 1 mg/L) [30,33]. -Median CT50 observed: 64.7 mg/L; median Cmin: 28.6 mg/L [30,33]. -Minimal ECMO effect; Vd/CL changes reflect sepsis/ARC/CRRT; AUC/t½ illness-driven. [30,33].	-Routine therapeutic drug monitoring (TDM) recommended, ≥50–100% fT > MIC (operational Cmin ≈1 mg/L, CT50 ≥ 4 mg/L); general ICU PK/PD threshold; adjustments are renal/CRRT-driven; no ECMO-specific dose changes required [30,33]. -Monitor renal function and clinical response regularly; adjustments primarily based on patient-specific factors [33].	*	-Suitable for empirical or targeted antibiotic therapy in critically ill ECMO patients, with no specific ECMO-related dosing adjustments generally necessary [30,33]. -Individualized dose adjustments guided by renal function and TDM recommended due to variability in critical illness [30,33].
Ceftazidime	-Standard dose (continuous infusion): Standard dosing: 2 g IV q8 h over 30 min. Extended infusion (3–4 h) or continuous infusion: LD 2 g IV, then 6 g/24 h (4 g/day if moderate renal impairment or continuous renal replacement therapy) [33,108]. -Ceftazidime/Avibactam (2.5 g IV q8h infused over 2 h, with renal adjustments): Clcr 30–50 mL/min: 1.25 g q8 h Clcr 16–30 mL/min: 0.94 g q12 h Clcr 6–15 mL/min or dialysis: 0.94 g q24 h [79,90].	-Minimal ECMO effect; Vd/CL shaped by renal status/CRRT; avibactam shows subexposure in ARC (AUC ↓); PK variability largely from renal function/CRRT. [5,79,108]. -Maintains therapeutic concentrations (>16 mg/L, ≥4 times the EUCAST breakpoint); general ICU PK goal; ECMO reports frequent sub exposure [79,90].	-Monitor plasma concentrations and clinical response, especially renal function [33,90]. -TDM recommended in prolonged therapy or significant renal impairment; adjustments are renal/CRRT-driven; ECMO effect minimal. [33,90].	*	-Empirical or targeted therapy for infections in critically ill ECMO patients [33,90]. -Dose adjustments primarily guided by renal function rather than ECMO itself [33,90]. -Continuous or extended infusion preferred to maintain concentrations above MIC for optimal pharmacodynamic efficacy [33,90].
Meropenem	-Standard dosing: Standard dosing: 1 g IV q8h over 30 min. [36]. Extended infusion (preferred): 1–2 g IV q8 h over 4 h [34,35]. -Continuous infusion: LD 2 g IV, then 3–6 g/24 h [34,35].	-Hydrophilic, low protein binding, minimal ECMO sequestration [5,35,36]. -ECMO rarely alters CL; mild ↑ Vd early; AUC/t½ largely illness-driven [5,35,36]. -Pharmacokinetic variability largely attributed to critical illness severity, renal function, patient weight, fluid balance, and concurrent renal replacement [5,35,36].	-TDM and individualized dose adjustments guided by plasma 100% fT > MIC (100% fT > 4 × MIC for severe infection); general ICU target; ECMO PopPK supports same goals; adjustments are renal/CRRT-driven [30,35,36,91,98]. -Increased likelihood of subtherapeutic concentrations in severe infections, patients on CRRT, or resistant pathogens [30,35,36,91,98].	Alternative carbapenems (e.g., imipenem-cilastatin), cefepime, piperacillin-tazobactam, or β-lactam/β-lactamase inhibitor combinations, considering pathogen susceptibility profile [35].	-Indicated for severe infections, especially with multi-drug-resistant pathogens [35,36,109]. -ECMO alone does not necessitate dosing adjustments; extended or continuous infusions strongly recommended [35,36,109]. -Close monitoring and individualized dose adjustments critical, primarily influenced by renal function and critical illness status [35,36,109].
Ertapenem	-No ECMO-specific PK data available. Standard dosing in critically ill patients typically recommended: 1 g IV q24 h over 30 min [5]. -Consider prolonged or continuous infusion strategies in patients receiving RRT [5]. -ECMO-specific dosing adjustments have not been clearly defined in the literature [5].	No ECMO-specific data about PK; dose adjustment should follow critical-illness principles, in practice renal/CRRT/illness drive Vd/CL/AUC/t½ [5].	-Regular clinical monitoring recommended. Adjustments follow renal/CRRT principles. -%fT > MIC ≥ 50–100% (extrapolated); target from general ICU; no ECMO-specific threshold [5].	Alternative carbapenems such as meropenem or imipenem-cilastatin for broader spectrum coverage, especially in severe infections or patients with ECMO-supported critical illness [5].	-Limited evidence available specifically for ECMO patients; use cautiously with consideration for renal replacement therapy [110,111,112]. -Adjust dosing strategies (prolonged or continuous infusion) primarily based on clinical condition and renal function, considering potential theoretical risk of ECMO sequestration [110,111,112].
Linezolid	-Standard dose: 600 mg IV q12 h over 30–60 min if MIC ≤ 1 mg/L [13,33,109]. -Higher dosing: Consider 600 mg IV q8h or continuous infusion: 1800 mg/24 h for pathogens with MIC > 1 mg/L [49,50,108].	↑ Vd (inflammation) with variable AUC/CL → frequent underexposure; ECMO adds variability but no consistent shift [49,50,108].	-Frequent TDM recommended with plasma concentration target between 2 and 7 mg/L to maintain therapeutic effectiveness and minimize toxicity risks, particularly thrombocytopenia; general ICU target; ECMO shows inconsistent exposures but no new window. [48,49,50,108]. -Individualize dosing based on measured concentrations and clinical response due to significant variability among ECMO patients [48,49,50,108].	Vancomycin or daptomycin (depending on clinical indication), or alternative agents based on susceptibility profiles and patient-specific factors [13,33,109].	-Indicated for severe Gram-positive bacterial infections. Due to high pharmacokinetic variability, ECMO patients frequently require individualized dosing beyond standard recommendations [48,49,50,108]. -Risk of subtherapeutic exposure and thrombocytopenia necessitates rigorous monitoring and possible dose increases, particularly for pathogens with MIC > 1 mg/L [48,49,50,108].
Vancomycin	-Loading dose: 25 mg/kg IV [53,54,55,99]. -Maintenance dose: 12.5–20 mg/kg IV q12 h [53,54,55,99]. -Alternative regimens [53,54,55,99]: 500 mg IV q8 h (lung transplant prophylaxis). 400 mg IV q8 h (if MIC ≤ 0.5 μg/mL). 600 mg IV q8 h (if MIC ≤ 1 μg/mL). -Renal-based adjustments [53,54,55,99]: Creatinine < 1 mg/dL: 1 g IV q8 h. Creatinine > 1 mg/dL: 1 g IV q12 h. Standard daily dose: 2 g/day divided q12 h.	-Minimal ECMO circuit sequestration risk [5,33]. -Vd/CL ≈ on vs. off ECMO; AUC variability high from renal function/CRRT; t½ variable. -Significant interindividual variability related primarily to critical illness, renal function, and renal replacement therapy rather than ECMO itself [30,59,60].	Regular monitoring of serum vancomycin trough levels recommended. AUC24/MIC 400–600 (surrogate trough 15–20 µg/mL if AUC unavailable); general ICU target; multiple ECMO PopPK confirm same goal [48,56,57,58,99].	*	-Broadly used for Gram-positive infections, particularly MRSA infections [53,56,57,58,99]. -Higher loading and maintenance doses, as well as continuous or prolonged infusion, recommended to achieve optimal AUC/MIC, especially if renal replacement therapy is used [53,56,57,58,99]. -Careful therapeutic monitoring required due to variability and potential nephrotoxicity risks [53,56,57,58,99].
Piperacillin-Tazobactam	-Extended infusion (preferred) dosing strategies based on renal function [30,92,113,114]: CrCl > 40 mL/min: 4.5 g IV q6 h over 4 h. CrCl 20–40 mL/min or continuous renal replacement therapy (CRRT): 4.5 g IV q8 h over 4 h. CrCl < 20 mL/min: 4.5 g IV q12 h over 4 h. -Consider higher loading doses and continuous infusion for severe infections, pathogens with elevated MIC values, or adequate renal function [93,98].	-Hydrophilic antibiotic, with low ECMO-related pharmacokinetic alterations [30,92,113,114]. -ECMO has small/no consistent effect; CL/Vd driven by renal function/CRRT; AUC/t½ illness-dependent. -Pharmacokinetic variability mainly influenced by patient-specific factors such as renal function, critical illness severity, and presence of renal replacement therapy rather than ECMO itself [30,92,113,114].	-Frequent TDM recommended, targeting plasma concentrations ≥ 64 mg/L for at least 50–100% of dosing; general ICU PK/PD target; ECMO adds variability [30,92,98,113,114]. -Rigorous monitoring is advised to prevent subtherapeutic or toxic exposure [30,92,98,113,114].	Meropenem, cefepime, carbapenems, or alternative β-lactam/β-lactamase inhibitor combinations based on susceptibility profiles, renal function, and clinical scenario [92,113,114].	-Choice for broad-spectrum coverage in ECMO-supported critically ill patients [30,92,98,113,114]. -Extended or continuous infusions strongly recommended [30,92,98,113,114]. -No ECMO-specific dosing adjustments generally required, but individual dosing should be guided by therapeutic monitoring, patient renal function, MIC values, and clinical response [30,92,98,113,114].
Tigecycline	-Standard dosing: LD 100 mg IV, then 50 mg IV q12 h over 30–60 min [5,13,97,109]. -Consider potential dose adjustment or monitoring for therapeutic effectiveness in critically ill ECMO patients due to theoretical ECMO circuit sequestration risk [5,109].	ECMO effect uncertain; exposure variability reflects illness/hepatic function; AUC changes inconsistent [5,109].	-Regular therapeutic and plasma concentration monitoring recommended to ensure therapeutic efficacy, particularly due to theoretical ECMO sequestration risk and high interindividual variability among critically ill patients [5,109]. -Software-assisted dosing and pharmacokinetic modeling tools strongly recommended [80].	Cefiderocol, colistin, or carbapenems as alternative antimicrobials for multi-drug-resistant Gram-negative bacterial infections [13,97].	-Indicated for severe infections including ventilator-associated pneumonia caused by Gram-negative bacilli and Staphylococcus epidermidis [5,13,97,109]. -Limited evidence specifically in ECMO; standard dosing appears effective, but careful therapeutic monitoring and individualized dosing strategies are advised due to potential pharmacokinetic variability [5,13,97,109].
Amphotericin B	Standard dose: 1 mg/kg/day IV [5,40,109]. No ECMO-specific dose adjustments generally required.	Minimal ECMO circuit effect; Vd/CL/AUC similar to non-ECMO; t½ unchanged [5,39,40,109].	Monitoring focuses on clinical efficacy and safety (renal function, electrolytes) [5,39,40,109].	*	-Indicated primarily for invasive fungal infections such as aspergillosis [5,39,40,109]. -Standard dosing is effective in ECMO-supported critically ill patients; no significant pharmacokinetic alterations requiring dose adjustment [5,39,40,109].
Liposomal Amphotericin B	-Standard dosing: 3–5 mg/kg/day IV, titrated upwards as needed (5–10 mg/kg/day) especially in aspergillosis and mucormycosis [5,39,40]. -Consider doubling doses if therapeutic response inadequate [39].	Circuit adsorption possible → AUC variability ↑, Vd apparent variable; CL not consistently ECMO-changed [5,39,40,43].	-Frequent plasma concentration monitoring recommended due to significant interindividual pharmacokinetic variability. -No serum PK target; monitor efficacy and renal/electrolyte safety; general ICU thresholds; ECMO may add adsorption.	Echinocandins (e.g., caspofungin), azoles (e.g., posaconazole, voriconazole) depending on susceptibility and clinical scenario [5,40,43].	Indicated for severe invasive fungal infections (aspergillosis, mucormycosis). ECMO therapy significantly influences pharmacokinetics, often requiring higher-than-standard dosing [5,39,40,43].
Caspofungin	-Standard dose: Loading dose: 70 mg IV once daily. Maintenance: 50–70 mg IV daily [5,48,81,82,94,95]. -Higher dosing (e.g., 100 mg/day) may be required in patients with high lean body mass (>80 kg) or infections caused by less susceptible organisms (e.g., Candida parapsilosis) [94,95].	ECMO-specific effect negligible; variability chiefly by body weight/severity/severity of illness; AUC stable [48,92].	-Target concentrations suggested [39]: Cmax: ~11.95 μg/mL. Cmin: ~3.73 μg/mL; thresholds from general antifungal literature. -Titrate dosing individually based on clinical response, patient weight, renal/hepatic function, and MIC of the causative organism [5,48,53,94].	Anidulafungin, micafungin, or amphotericin B formulations depending on pathogen susceptibility, patient-specific conditions, and response to caspofungin therapy [5,40].	-Indicated primarily for invasive candidiasis in critically ill patients undergoing ECMO [5,48,53,94]. -Standard dosing is usually sufficient; higher dosing may be required due to interindividual variability and critical illness severity. ECMO itself does not generally require dosing adjustments [5,48,53,94].
Fluconazole	-Standard dose: Loading dose 12 mg/kg IV, maintenance 6 mg/kg/day IV if MIC ≤ 1 mg/L [39,83,115]. -Intensified dose: Loading dose 18 mg/kg IV, maintenance 6 mg/kg/day if MIC 1–2 mg/L [115]. -High-intensity dose: Loading dose 18 mg/kg IV, followed by maintenance 12 mg/kg/day or 6 mg/kg every 12 h if MIC ≥ 2 mg/L [115]. -Alternative fixed daily dosing: 400–800 mg/day for MIC ≤ 1 mg/L infections (e.g., Candida albicans) [48].	ECMO effect minimal; AUC/CL governed by renal function (±CRRT); Vd ↑ modestly; t½ renal-dependent; may necessitate higher loading doses especially for pathogens with higher MIC values (≥2 mg/L) [39,82,83].	-Target trough 10–15 mg/L (or AUC/MIC ≥ 100 if available). Check levels especially in CRRT or high MIC isolates; escalate dose or shorten interval if <10 mg/L; general ICU target [82]. -Individualized monitoring and dose titration according to clinical response advised [82].	Voriconazole, caspofungin, liposomal amphotericin B, or echinocandins depending on antifungal susceptibility profile, clinical scenario, and patient-specific considerations [39,83].	-Effective in ECMO-supported patients with minimal sequestration risk, but dosing adjustments (primarily higher loading doses) may be required due to increased volume of distribution in ECMO therapy or renal replacement therapy [39,48,82,83]. -Standard dosing generally adequate if MIC ≤1 mg/L, but individualized therapeutic monitoring strongly recommended [39,48,82,83].
Voriconazole	-IV dosing: Loading dose 6 mg/kg every 12 h (first 24 h), maintenance dose 4 mg/kg every 12 h [39,53,83]. -PO dosing: Loading dose 400 mg every 12 h (first 24 h), maintenance dose 200 mg every 12 h [47]. -Due to significant variability, maintenance dose often increased to ≥ 6 mg/kg every 12 h, and up to 12 mg/kg every 12 h in subtherapeutic (<2 mg/L) cases [39,44,45,46,53,83].	Substantial sequestration in ECMO circuits, causing significant interpatient pharmacokinetic variability and subtherapeutic concentrations at standard dosing, possible subexposure (AUC ↓, troughs < 2 mg/L); Vd ↑; CL variable; t½ variable [39,53,83].	-Target trough (Cmin) 2–5 mg/L; general ICU therapeutic window; ECMO confirms frequent subexposure [40,47,48,109]. -Stepwise escalation in ECMO: 1. Confirm adequate loading (6 mg/kg IV q12h × 2 doses). 2. If trough < 2 mg/L on standard maintenance (4 mg/kg IV q12h), increase maintenance by 25–50% (e.g., 5–6 mg/kg q12h) and recheck level after 4–5 doses. 3. If still subtherapeutic (<2 mg/L) despite escalated dosing, consider switching: liposomal amphotericin B if broad mold coverage required, or an echinocandin (e.g., caspofungin) in case of intolerance or resistant Candida.	Alternative antifungals: Liposomal amphotericin B, isavuconazole, posaconazole, or echinocandins (caspofungin, anidulafungin), depending on pathogen susceptibility, renal/hepatic function, and ECMO-related pharmacokinetic considerations [13,40,48].	Standard antifungal agent for invasive aspergillosis or other fungal infections in critically ill ECMO-supported patients. Significant variability and ECMO sequestration often necessitate increased dosing and individualized therapeutic drug monitoring. [13,39,40,48,83,92,109].
Epinephrine	-General recommendation: Use minimal effective doses (0.5–1 μg/kg/min), titrated based on clear perfusion targets (SvO_2_ >60%, lactate < 6 mmol/L, cardiac index >2.2 L/min/m^2^) [66,84]. -ECPR-specific: Limit cumulative dose to ≤3 mg total to improve neurological outcomes and hospital survival. Avoid cumulative doses > 3 mg due to increased risk of adverse effects [64].	-No consistent ECMO effect on Vd/CL/AUC/t½; dose–response reflects illness severity; observational harm signal likely confounding by indication [60,61,62,94]. -Increased myocardial oxygen consumption, hyperlactatemia, splanchnic vasoconstriction, immunosuppressive effects, and potential microvascular cerebral ischemia at higher cumulative doses [64,65,66,84]. -Prioritize goal-directed endpoints (e.g., MAP ≥ 65 mmHg, CI > 2.2 L/min/m^2^, lactate down-trending, adequate SvO_2_) to minimize dose escalation; reserve primarily as rescue agent [64,65,66,84].	Rigorous monitoring of hemodynamic parameters (MAP, cardiac index, SvO_2_), lactate levels, and clinical response. Individualized titration critical to minimize risks; general ICU practice; ECMO has no numeric PK goal [66,84].	-Prefer alternative agents such as inodilators (dobutamine, milrinone, levosimendan) or norepinephrine for maintaining hemodynamic stability [65,66,84]. -Epinephrine recommended primarily as rescue therapy in refractory shock or cardiac arrest situations [65,66,84].	-Strongly discourage routine use due to association with increased mortality and significant adverse effects [64,65,66,84]. -Recommended as second-line or rescue therapy under careful monitoring. Strict adherence to minimal effective dosing and defined perfusion targets necessary to optimize patient outcomes [64,65,66,84].
Dobutamine	Avoid doses >5 μg/kg/min once mean arterial pressure (MAP) ≥ 65 mmHg is achieved, as higher doses provide no additional therapeutic benefit [66].	-PK changes driven by patient’s cardiac function and illness severity, not ECMO [66]. -Use the lowest effective dose and titrate to goal-directed endpoints (CI > 2.2 L/min/m^2^, MAP ≥ 65 mmHg, evidence of adequate perfusion), considering inodilator alternatives when appropriate to avoid unnecessary up-titration [66]. -Catecholamine-based inotrope; prolonged or excessive use may increase the risk of arrhythmias and cardiomyopathy, especially at doses above 5 μg/kg/min after hemodynamic stabilization [66].	-Monitor MAP (≥65 mmHg), cardiac index (>2.2 L/min/m^2^), and mixed venous oxygen saturation (SvO_2_ > 60%); general ICU practice; no ECMO PK threshold [66]. -Regularly assess cardiac rhythm and function to promptly detect arrhythmias or signs of cardiotoxicity [66].	Inodilators such as milrinone or levosimendan preferred due to superior safety profile and beneficial effects on myocardial contractility and perfusion [66].	-Dobutamine is suitable for short-term hemodynamic stabilization [66]. -Limit dosing and duration to avoid adverse cardiac effects. Alternative inodilators are preferred in stable patients to minimize risk of cardiotoxicity and arrhythmias [66].
Norepinephrine	-Standard dosing: 0.03–0.30 μg/kg/min. Typical max: 0.22–0.30 μg/kg/min [61,63]. -Moderate-high dosing: (0.5–1 μg/kg/min, up to 100 μg/min) reported in severe hypotension or septic cardiomyopathy, titrated carefully according to clinical response [62,84].	-No significant ECMO-related pharmacokinetic changes or sequestration risk were observed [61,62,63,84]. -Dose variability primarily driven by critical illness severity, sepsis, myocardial dysfunction, vasoplegia, so emphasize goal-directed endpoints (MAP ≥ 65 mmHg, CI > 2.2 L/min/m^2^, lactate/SvO_2_ targets) rather than escalating for ECMO needs [61,62,63,84].	-Regular monitoring recommended, titrating to clear hemodynamic targets: MAP ≥ 65 mmHg, cardiac index > 2.2 L/min/m^2^, SvO_2_ > 60%, lactate levels < 6 mmol/L, and clinical response; general ICU target [62,84]. -ECMO flow rate impacts cardiac overload and pulmonary congestion, emphasizing careful ECMO flow management [61].	Epinephrine (as rescue), vasopressin, or inodilators such as levosimendan, milrinone, or dobutamine for cardiac support or hemodynamic stabilization in specific clinical scenarios [63].	-First-line vasopressor for refractory hypotension and septic cardiomyopathy in critically ill ECMO patients [61,62,63,84]. -Dosage and titration guided by hemodynamic response and perfusion goals. High-dose norepinephrine should be carefully justified due to associated risks and severity of clinical condition, rather than ECMO-specific pharmacokinetics [61,62,63,84].
Unfractionated Heparin (UFH) ¤	-IV bolus at cannulation: 50–100 IU/kg, followed by continuous infusion 10–21 IU/kg/h adjusted to therapeutic goals [13,66,67]. -Typical ECMO dosages: ECMO-VA: ~9.59 IU/kg/h (approx. 8500 IU/day). ECMO-VV: ~13.64 IU/kg/h (approx. 28,800 IU/day) [96].	-High molecular weight (~3000–30,000 Da) and hydrophilic nature limit significant ECMO circuit sequestration [5]. -Pharmacokinetics largely influenced by critical illness severity, renal function, body weight, inflammation, and concurrent renal replacement therapy (CRRT) rather than ECMO itself [69,116].	-ECMO practice specific monitoring ranges: aPTT: 50–90 s Anti-Factor Xa (FXa): 0.3–0.7 IU/mL (preferred due to lower variability) ACT: 140–220 s [13,66,67]. -Multimodal monitoring (FXa combined with viscoelastic testing ROTEM/TEG) strongly recommended for precise dosing adjustments [13,66,67].	-Bivalirudin (0.1–0.15 mg/kg/h IV infusion) or Argatroban (0.1–0.5 mg/kg/min IV infusion) recommended alternatives in patients developing heparin-induced thrombocytopenia (HIT), especially with hepatic or renal impairment respectively [13,66,67]. -Low molecular weight heparin (LMWH; enoxaparin 0.5 mg/kg twice daily SC) as viable alternative in selected patients [86].	-Anticoagulation standard of care in ECMO-supported patients. -Dose individualization and rigorous therapeutic monitoring crucial due to high interindividual pharmacokinetic variability. Limit dosing to the lowest effective anticoagulation level to balance thrombotic and hemorrhagic risks. Special caution and monitoring for HIT recommended [13,66,67,68,69].
Enoxaparin	-Prophylactic dosing: 0.5 mg/kg SC twice daily (8000 IU/day divided into two doses of 4000 IU every 12 h), target peak FXa levels: 0.3–0.5 IU/mL [73]. -IV continuous infusion: Loading dose 0.5 mg/kg IV prior to cannulation, followed by continuous infusion at 1 μg/kg/min (≈10 IU/kg/min), target FXa: 0.4–0.6 IU/mL [74].	No clear ECMO-specific effect. Effective anticoagulant with stable and predictable pharmacodynamic profile in critically ill ECMO patients [5,73,74].	-Therapeutic monitoring via anti-FXa levels strongly recommended in ECMO practice (target peak: 0.3–0.6 IU/mL) [67,73,74]. -Prefer FXa monitoring over aPTT or ACT for more precise dosing and minimizing bleeding risk [67,73,74].	*	-Preferred anticoagulant in ECMO patients contraindicated for UFH or requiring simpler administration [67,73,74]. -Demonstrates lower incidence of thromboembolic and bleeding events compared to UFH. IV continuous infusion guided by FXa monitoring considered safe and effective but requires further clinical evidence [67,73,74].
Rocuronium	Standard neuromuscular blockade dosing (No ECMO-specific adjustments available) [77,78,117].	-Potential early circuit adsorption (↑ Vd); later accumulation reflects hepatic dysfunction; CL ↓ if liver failure; t½ may ↑ [22]. -Rocuronium has potential ECMO-related adsorption risk due to high lipophilicity [22].	Monitor clinical response closely, particularly neuromuscular function and hepatic status. No specific ECMO-related TDM available.	Cisatracurium preferred due to lower lipophilicity, rapid onset of action, and metabolism independent of hepatic and renal pathways [22].	Valid neuromuscular blocker in ECMO-supported patients, but risk of active metabolite accumulation in hepatic impairment limits its optimal use [22].

**Figure A1:**
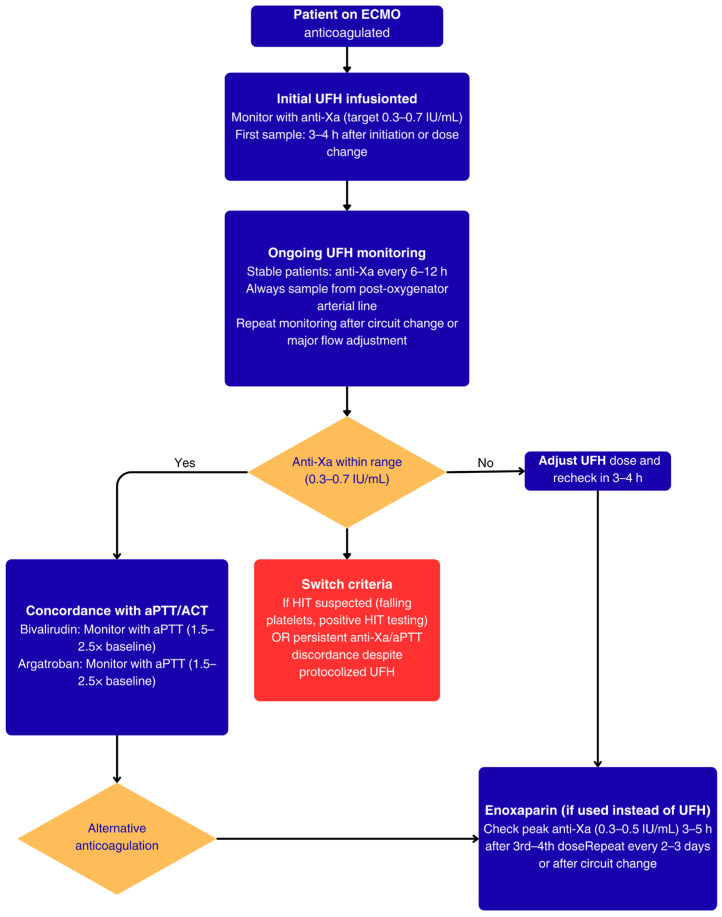
Anticoagulation monitoring pathway in ECMO patients.

Protein binding was categorized as low (<30%), moderate (30–70%), and high (>70%). Lipophilicity was defined by logP: low (<1), moderate (1–2), and high (>2). Abbreviations: ARC, Augmented Renal Clearance; INN, International Nonproprietary Name; PB, Protein Biding; PK/PD, Pharmacokinetic/Pharmacodynamic; TDM, Therapeutic Drug Monitoring; IV, Intravenous; BMI, Body Mass Index; MIC, Minimum Inhibitory Concentration; ECMO, Extracorporeal Membrane Oxygenation; VV-ECMO, Veno-venous ECMO; VA-ECMO, Veno-arterial ECMO; RASS, Richmond Agitation-Sedation Scale; CPOT, Critical-Care Pain Observation Tool; aPTT, Activated Partial Thromboplastin Time; ACT, Activated Clotting Time; FXa, Anti-factor Xa; SvO_2_, Mixed Venous Oxygen Saturation; MAP, Mean Arterial Pressure; CRRT, Continuous Renal Replacement Therapy; KDIGO, Kidney Disease Improving Global Outcomes; MDR, Multi-Drug Resistant; MRSA, Methicillin-resistant Staphylococcus aureus; PRIS, Propofol Infusion Syndrome; HIT, Heparin-Induced Thrombocytopenia; BPS, Behavioral Pain Scale. * No relevant or sufficient data was identified in the scientific literature, based on the predefined search strategy and inclusion/exclusion criteria used to make a suggest or recommendation. ¤ Detailed monitoring steps for anticoagulation (UFH, enoxaparin, and direct thrombin inhibitors) are illustrated in Figure A1. Table 8 summarizes only the key therapeutic drug monitoring targets. Directional arrows indicate the qualitative change of pharmacokinetic parameters relative to non-ECMO conditions: ↑ = increase, ↓ = decrease, ≈ = no consistent change.

**Table 9:** Relationship between physicochemical properties and exposure changes in adult ECMO patients.

Drug	logP	Protein Binding (%)	ECMO Effect on Plasma Concentrations (Clinical Adult Data)
Midazolam	3.3	95–97%	Higher dose requirements; lower plasma concentrations vs. expected. No % quantified in adult cohorts [12,88].
Fentanyl	4.0	80–85%	Increased early dose requirements; lower levels reported. No % quantified in adult cohorts [16,18,85].
Propofol	3.8	>95%	Increased dose needs; lower concentrations observed. No % quantified in adult cohorts [5,14].
Voriconazole	2.6	>95%	Subtherapeutic troughs (<2 mg/L) in ~30–50% of ECMO patients [44,45,46,47].
Posaconazole	3.0	>98%	Subtherapeutic levels frequent; ~40% below prophylaxis target in ECMO patients [39].
Fluconazole	0.5	10–12%	Stable exposures; no significant reduction in ECMO vs. non-ECMO [115].
Meropenem	–0.2	<2%	Comparable PK on/off ECMO; no significant loss observed [36,91].
Amikacin	–5.6	<5%	Subtherapeutic peaks frequent with standard LD; ECMO not independent predictor. No % quantified [29,32,48].
Vancomycin	–3.1	30–55%	No consistent ECMO effect; AUC variability renal/CRRT-driven [54,55,58].

Only clinical adult ECMO data were included. Percentages of subtherapeutic exposures are reported when available (e.g., voriconazole, posaconazole). For sedatives and several antimicrobials, adult studies describe higher dose requirements or lower plasma concentrations, but without quantifiable % of sequestration; such values have been excluded to avoid reliance on ex vivo data.

## Data Availability

No new data were created or analyzed in this study.

## Abbreviations

The following abbreviations are used in this manuscript:

Abbreviation	Definition
ACT	Activated Clotting Time
ARC	Augmented Renal Clearance
AUC	Area Under the Curve
BMI	Body Mass Index
BPS	Behavioral Pain Scale
CI	Cardiac Index/Continuous Infusion (specify in context)
CL	Clearance
CPOT	Critical-Care Pain Observation Tool
CRRT	Continuous Renal Replacement Therapy
ECMO	Extracorporeal Membrane Oxygenation
EI	Extended Infusion
FXa	Anti-factor Xa
HIT	Heparin-Induced Thrombocytopenia
ICU	Intensive Care Unit
INN	International Nonproprietary Name
IV	Intravenous
KDIGO	Kidney Disease Improving Global Outcomes
LD	Loading Dose
MAP	Mean Arterial Pressure
MDR	Multi-Drug-Resistant
MIC	Minimum Inhibitory Concentration
MRSA	Methicillin-Resistant Staphylococcus aureus
PK	Pharmacokinetics
PD	Pharmacodynamics
PopPK	Population Pharmacokinetics
PRIS	Propofol Infusion Syndrome
RASS	Richmond Agitation-Sedation Scale
SvO_2_	Mixed Venous Oxygen Saturation
TDM	Therapeutic Drug Monitoring
UFH	Unfractionated Heparin
VA-ECMO	Veno-arterial ECMO
Vd	Volume of Distribution
VV-ECMO	Veno-venous ECMO
t½	Elimination half-life

## Appendix A

**Table A1:** Selected databases, keywords, search strategies, and filters used in the initial article retrieval process.

Date Base	Search Strategy	Filters Applied in the Datebase	Date of Last Searched
PubMed/MEDLINE	(“Extracorporeal Membrane Oxygenation” [Mesh] OR ECMO OR “Extracorporeal Life Support”) AND (“Intensive Care Units” [Mesh] OR “Critical Care” [Mesh] OR ICU OR “Intensive Care”) AND (“INN” [Mesh]) AND (“Pharmacokinetics” [Mesh] OR pharmacokinetics OR “Dose-Response Relationship, Drug” [Mesh] OR “Dose Titration” OR therapeutic monitoring) AND (“Adult” [Mesh] OR adult OR “young adult” [Mesh] OR “middle aged” [Mesh] OR “Aged” [Mesh]) “INN dose adjustment on ECMO” “INN pharmacokinetics on ECMO”	Clinical Study, Clinical Trial, Guideline, Meta-Analysis, Observational Study, Randomized Controlled Trial, Review, Systematic Review, in Adult: 19+ years, published in the Last 10 Years.	11 May 2025
EMBASE	(‘extracorporeal membrane oxygenation’/exp OR ECMO OR ‘extracorporeal life support’) AND (‘intensive care unit’/exp OR ICU OR ‘critical care’ OR ‘intensive care’) AND (‘INN’) AND (‘pharmacokinetics’/exp OR ‘pharmacodynamics’/exp OR ‘dose titration’/exp OR ‘therapeutic drug monitoring’/exp) “INN dose adjustment on ECMO” “INN pharmacokinetics on ECMO”	Articles, Review Articles and clinical trials published in the Last 10 Years	11 May 2025
Scopus	(“extracorporeal membrane oxygenation” OR ECMO OR “extracorporeal life support”) (“intensive care” OR ICU OR “critical care”) (“INN”) (“pharmacokinetics” OR “pharmacodynamics” OR “dose titration” OR “therapeutic monitoring”) (“adult” OR “patients over 18 years”) “INN dose adjustment on ECMO” “INN pharmacokinetics on ECMO”	Articles and Review Articles published in the Last 10 Years	11 May 2025
Cochrane Library	“INN” AND “ECMO”	Clinical Trials, Observational Studies, Systematic Reviews, and Meta-analyses published in the Last 10 Years.	11 May 2025
Sage Journals	“INN AND Dose Adjustment AND Pharmacokinetics AND Pharmacodynamics AND Extracorporeal Membrane Oxygenation AND Adults”	Research Articles and Review Articles published in the Last 10 Years	11 May 2025
ScienceDirect	“INN AND Dose Adjustment AND Pharmacokinetics AND Pharmacodynamics AND Extracorporeal Membrane Oxygenation AND Adults”	Research Articles, Review Articles and Practice Guidelines published in the Last 10 Years	11 May 2025
Taylor & Francis Online	“INN AND Dose Adjustment AND Pharmacokinetics AND Pharmacodynamics AND Extracorporeal Membrane Oxygenation AND Adults”	Articles published in the Last 10 Years	11 May 2025
SpringerLink	“INN AND Dose Adjustment AND Pharmacokinetics AND Pharmacodynamics AND Extracorporeal Membrane Oxygenation AND Adults”	Articles, Research Articles, Review Articles and Practice Guidelines published in the Last 10 Years	11 May 2025
Specialized clinical databases (Micromedex, DynaMed, UpToDate)	INN	N.A.	11 May 2025
